# Tumor cells derived exosomes contain hTERT mRNA and transform nonmalignant fibroblasts into telomerase positive cells

**DOI:** 10.18632/oncotarget.10384

**Published:** 2016-07-02

**Authors:** Anna Gutkin, Orit Uziel, Einat Beery, Jardena Nordenberg, Maria Pinchasi, Hadar Goldvaser, Steven Henick, Michal Goldberg, Meir Lahav

**Affiliations:** ^1^ The Felsenstein Medical Research Center, Rabin Medical Center, Petah Tikva, Israel; ^2^ Sackler School of Medicine, Tel-Aviv University, Petah Tikva, Israel; ^3^ Institute of Oncology, Davidoff Cancer Center, Rabin Medical Center, Petah Tikva, Israel; ^4^ Department of Genetics, Institute of Life Sciences, Hebrew University of Jerusalem, Petah Tikva, Israel; ^5^ Institute of Hematology, Davidoff Cancer Center, Rabin Medical Center, Petah Tikva, Israel

**Keywords:** exosomes, telomerase

## Abstract

Exosomes are small (30-100nm) vesicles secreted from all cell types serving as inter-cell communicators and affecting biological processes in “recipient” cells upon their uptake. The current study demonstrates for the first time that hTERT mRNA, the transcript of the enzyme telomerase, is shuttled from cancer cells via exosomes into telomerase negative fibroblasts, where it is translated into a fully active enzyme and transforms these cells into telomerase positive, thus creating a novel type of cells; non malignant cells with telomerase activity. All tested telomerase positive cells, including cancer cells and non malignant cells with overexpressed telomerase secreted exosomal hTERT mRNA in accordance with the endogenous levels of their hTERT mRNA and telomerase activity. Similarly exosomes isolated from sera of patients with pancreatic and lung cancer contained hTERT mRNA as well. Telomerase activity induced phenotypic changes in the recipient fibroblasts including increased proliferation, extension of life span and postponement of senescence. In addition, telomerase activity protected the fibroblasts from DNA damage induced by phleomycin and from apoptosis, indicating that also telomerase “extracurricular” activities are manifested in the recipient cells. The shuttle of telomerase from cancer cells into fibroblasts and the induction of these changes may contribute to the alterations of cancer microenvironment and its role in cancer. The described process has an obvious therapeutic potential which will be explored in further studies.

## INTRODUCTION

Telomerase, a unique reverse transcriptase maintains telomere length by synthesizing TTAGGG repeats at their ends [1]. In humans telomerase is transcriptionally repressed at birth and remains inactive through life in most somatic cells. Telomerase activity prevents cellular replicative senescence and confers longevity or even immortality to the cell. It has several cellular functions, some of them are telomere related and some, e.g. prevention of apoptosis and others are independent of telomere length maintenance and termed “extracurricular” activities of telomerase [2]. Telomerase repression in human somatic cells is a major cause of senescence, aging and as such is considered a cancer suppressive mechanism. However, telomerase is activated in >90% of cancer cells and considered *a hallmark of cancer.* Its activity is essential for the endless proliferation and the perpetuation of the malignant clone [3]. Several recent studies so far demonstrated that transcripts of telomerase (hTERT, human telomerase reverse transcriptase) can be detected in the serum of cancer patients in breast, colon, hepatocellular carcinoma and follicular lymphoma([4 and references therein).

Exosomes are small (30-100nm) membrane vesicles that originate from the endosomal membrane compartment [5]. They contain mRNA, miRNA, DNA, long non coding RNA, proteins and lipids [6] and are secreted by many cell types into the microenvironment, therefore are detected in all kinds of body fluids. Likewise, cancer cells release exosomes into the tumor microenvironment and peripheral blood [7]. Exosomes are taken up by other cells, thus serving as mediators of cell to cell crosstalk. Upon transfer to recipient cells they can alter cell's molecular profile, signaling pathways and gene regulation [8].

The role of the cancer microenvironment in the perpetuation, expansion and aggressiveness of the malignant clone is well established [9]. Likewise, tumor cells maneuver the cancer microenvironment to support cancer progression and metastasis by influencing stromal cells and the extra cellular matrix. These processes are mediated by intercellular communications carried out among others by exosomes [10].

Accumulating data point to the various roles of exosomes secreted from cancer cells in the microenvironment. These include: promoting tumor cell growth and proliferation [11–14] and inducing angiogenesis [15, 16]. In addition, cancer derived exosomes are able to transform fibroblasts to cancer associated fibroblasts that typically support the tumor growth, vascularization and metastasis [17]. An addition layer of support is given by exosomes modification of the extracellular matrix [18–23]. Interestingly, these processes are not restricted to the immediate cancer surroundings but may also affect distant organs by exosomes secreted into body fluids [24–27]. Many articles describe various changes initiated by exosomal transfer; no research yet studied the “telomerase connection” between the telomerase positive cancer cells on telomerase negative somatic cells via exosomal cross talk.

In the current study we have characterized the secretion of hTERT mRNA by cancer cells derived exosomes. We show that all examined cancer cells secrete hTERT mRNA via exosomes. Exosomal hTERT mRNA concentration correlates with the telomerase activity and its expression in the cell of origin. hTERT mRNA is taken up by normal (telomerase negative) fibroblasts and undergoes translation and posttranslational processing rendering those cells telomerase positive. Our results describe the effects of induction of telomerase activity in previously telomerase negative fibroblasts. The transfer of telomerase mRNA significantly changed several cellular properties of the fibroblasts, such as proliferation rate, postponement of senescence, resistance to DNA damage and to apoptosis.

## RESULTS

### Exosomes derived from cancer cell lines, serum of cancer patients and hTERT transfected primary fibroblasts contain hTERT mRNA

**Figure 1:**
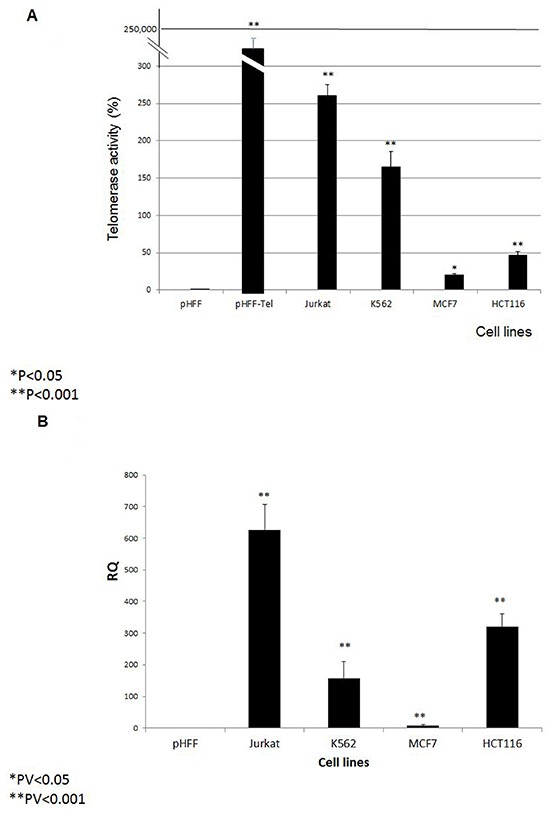
Intracellular hTERT mRNA and telomerase activity Four cancer cell lines (Jurkat, K562, MCF7 and HCT116), primary fibroblasts (pHFF cells) and primary fibroblasts transfected with the hTERT ORF (pHFF-Tel) were grown and analyzed for: **A**. Relative telomerase activity by the Q-TRAP assay and **B**. hTERT mRNA expression by quantitative real time PCR. The bars represent levels of each sample ± S.E of three or more independent experiments conducted in triplicates. * Indicates P<0.05, ** Indicates P<0.001.

Prior to exosome isolation, the relative telomerase activity and hTERT expression were demonstrated in the following cells: Jurkat (T cell leukemia), MCF-7 (breast carcinoma), K562 (chronic myeloid leukemia) and HCT116 (colon carcinoma); pHFF (primary fibroblasts cells which lack telomerase activity) and pHFF-Tel cells transfected with the hTERT gene. As expected, all cancer cells or cells in which telomerase was ectopically expressed presented telomerase activity, albeit at different levels (Figure 1A). These levels correlated with the expression of the hTERT gene (Figure 1B).

**Figure 2:**
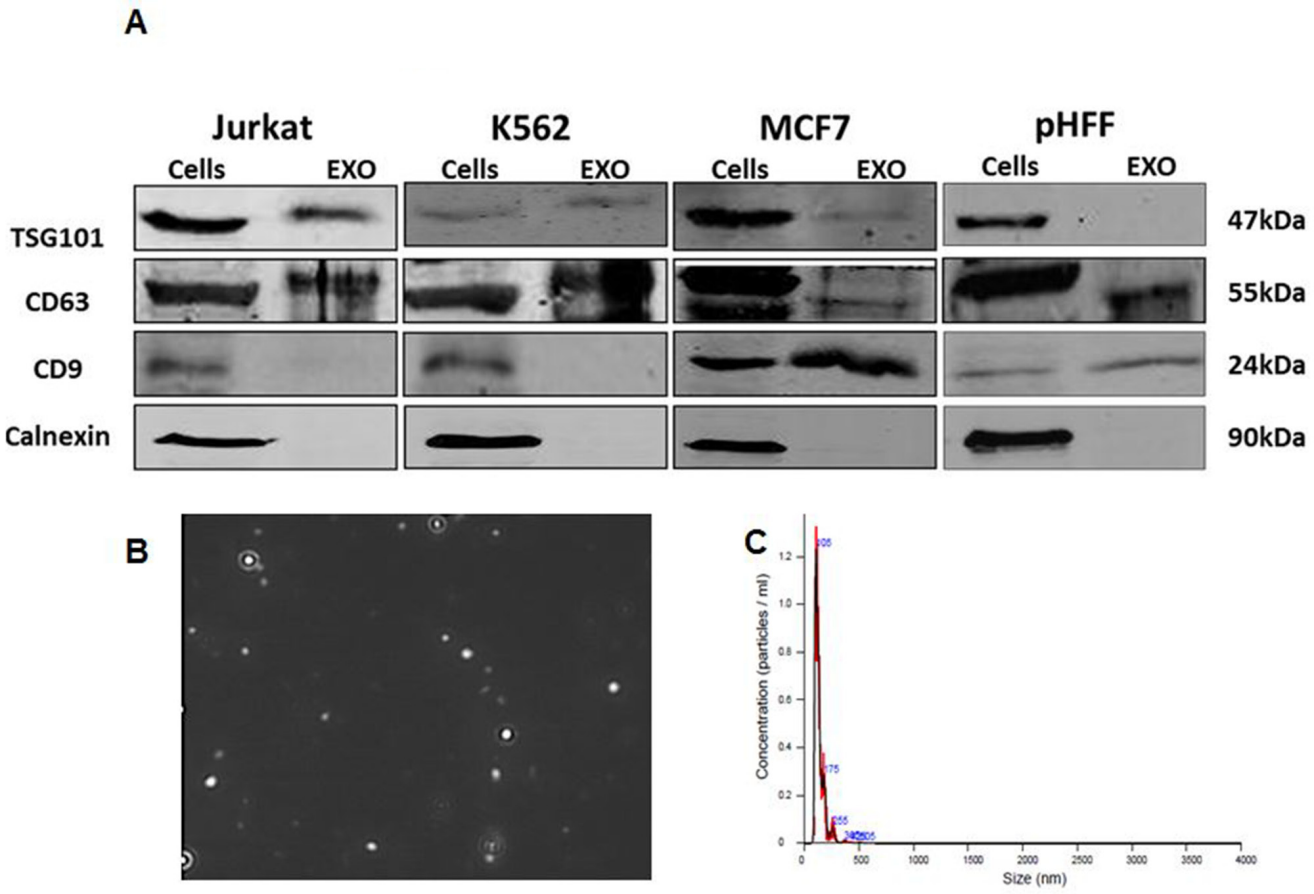
Validation of exosome isolation Exosomes were isolated from Jurkat, K562, MCF7 and HCT116 cells growth medium depleted from FBS exosomes as described in the Methods section following 72 hours of growth. **A**. Exosomal markers from all cells lines analyzed by Western blotting; **B.** Capture imaging of Jurkat exosomes obtained by using the NanoSight device; **C.** amount of these exosomes obtained specified by the NanoSight device.

Exosomes were isolated from the growth media of the four cell lines. The purity of the exosomes was verified by assessing the presence of specific exosomal markers (*e.g.* TSG101, CD9 and CD63) and the lack of non-exosomal marker (calnexin) by Western blot analysis in Jurkat cells (Figure 2A). As expected, calnexin was detected only in total cellular lysates and not in exosomes, indicating that our exosomal fractions are free of cellular components and debris. Furthermore, both TSG101 and CD63 were detected in exosomes derived from all 3 different cancer cell lines (Figure 2A). Figure 2B, 2C depicts the image of the Jurkat cells exosomes presented by the NanoSight imaging system.

**Figure 3:**
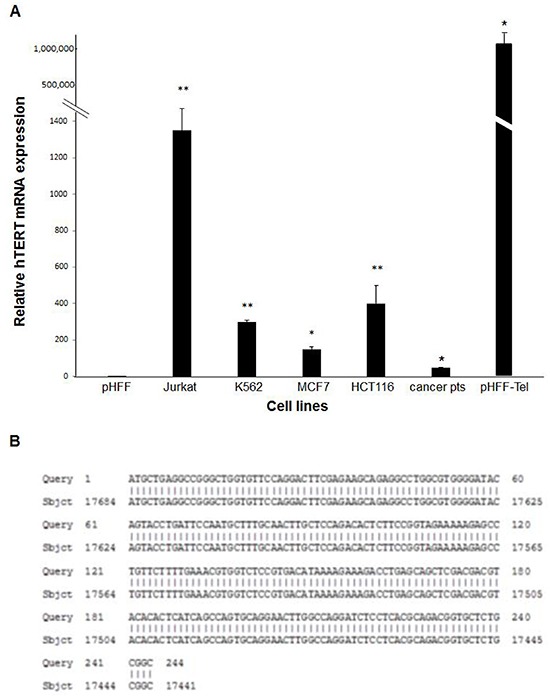
Cancer cells or non-cancer telomerase expressing cells secrete hTERT mRNA in their exosomes **A.** Levels of hTERT mRNA in various cancer cells, cancer patients' sera or non-cancer telomerase expressing cells. All cells were grown in a medium depleted exosomes. After 72 hours exosomes were isolated and hTERT mRNA levels were analyzed by quantitative real time PCR. The bars represent levels of hTERT mRNA of each sample ± S.E of three or more independent experiments conducted in triplicates. * Indicates P<0.05, ** Indicates P<0.001. **B**. Sequencing of the hTERT mRNA PCR product. The exosomal hTERT mRNA was amplified by PCR, sequenced and compared to the DNA database by Blast software.

Exosomes were isolated also from sera of 5 patients with active pancreatic or lung cancer. The levels of the hTERT mRNA were measured by q-PCR in exosomes secreted from the growth media of cancer cell lines, cancer patients' sera and telomerase negative and positive pHFF cells. All cancer cells derived exosomes contained hTERT mRNA albeit at different levels (Figure 3A). The level of hTERT mRNA in Jurkat cells derived exosomes was significantly higher compared to those obtained from the other cells; therefore we used these exosomes for further studies. Sequence analysis of the obtained PCR products and BLAST comparison verified the identity of the amplified products as hTERT mRNA (Figure 3B).

### Exosomal hTERT mRNA correlates with the intracellular hTERT mRNA and telomerase activity

**Figure 4:**
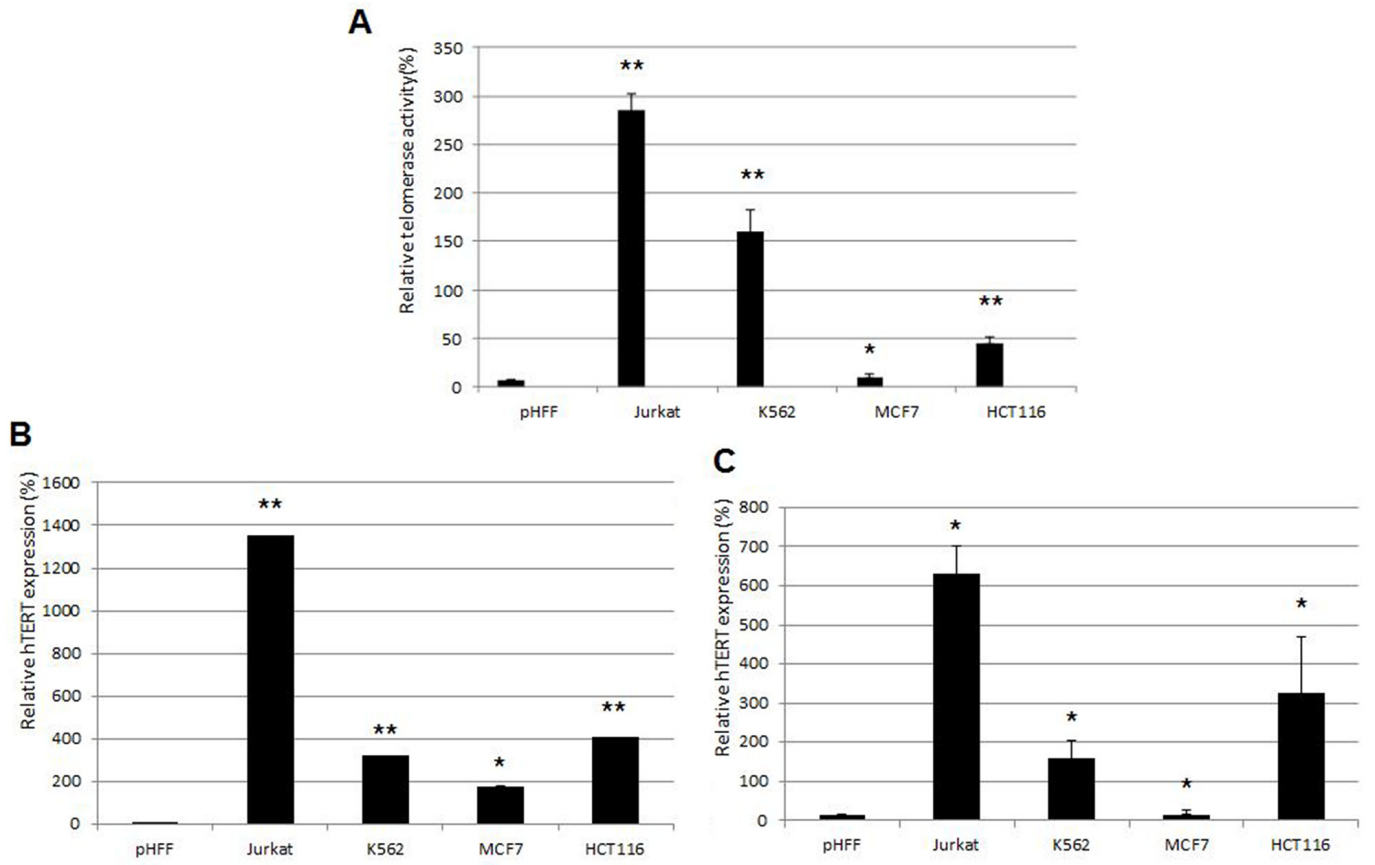
Correlations between telomerase activity in cancer and non cancer cells, hTERT mRNA expression and in their corresponding exosomes Jurkat, K562, MCF-7, HCT116 and pHFF cells were grown in exosomes free media for 72 hours. **A.** telomerase activity in cancer cell lines as assessed by the Q-TRAP assay; **B.** hTERT mRNA expression in cancer cell lines as measured by Q-PCR; **C.** hTERT mRNA expression in exosomes derived from the cancer cell lines as measured by Q-PCR.

We examined a possible correlation between the extracellular and intracellular hTERT mRNA levels and telomerase activity. As shown in Figure 4, telomerase activity in cancer cells was directly correlated with the levels of the intracellular hTERT mRNA levels and the level of exosomal hTERT mRNA. Jurkat cells exhibited the highest level of intracellular and extracellular hTERT mRNA as well as telomerase activity, whereas in MCF-7 cells these parameters were substantially lower compared to those obtained with other cells (Figure 1A, 1B; 3A). Therefore, Jurkat cells were chosen for the further continuation of the study. pHFF cells were devoid of telomerase activity and there was no hTERT expression neither in the cells nor in their exosomes. Obviously we could not correlate the exosomal hTERT levels with tumor telomerase activity in the patients' samples.

These results demonstrate that the levels of exosomal hTERT mRNA reflect the intracellular levels of telomerase activity and hTERT mRNA transcript, implying a strict regulation of this process.

### Exosomes from Jurkat cells are taken up by fibroblast cells and induce the expression and activity of telomerase in the recipient cells

**Figure 5:**
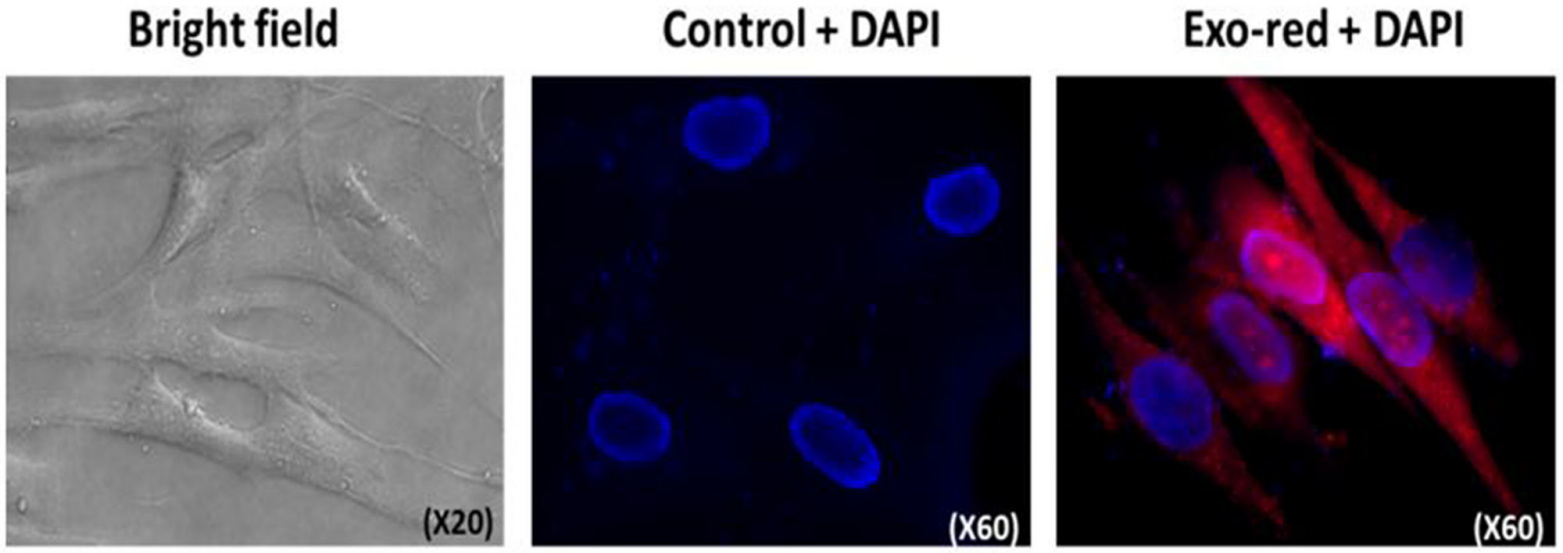
Jurkat cells derived exosomes are taken up by pHFF cells pHFF cells were incubated with Exo-red labeled exosomes derived from Jurkat cells' growth media for 6hr at 37^°^C, stained with DAPI and analyzed by fluorescent microscopy. Exosomes are shown after penetration to the fibroblasts in red at the right panel.

**Figure 6:**
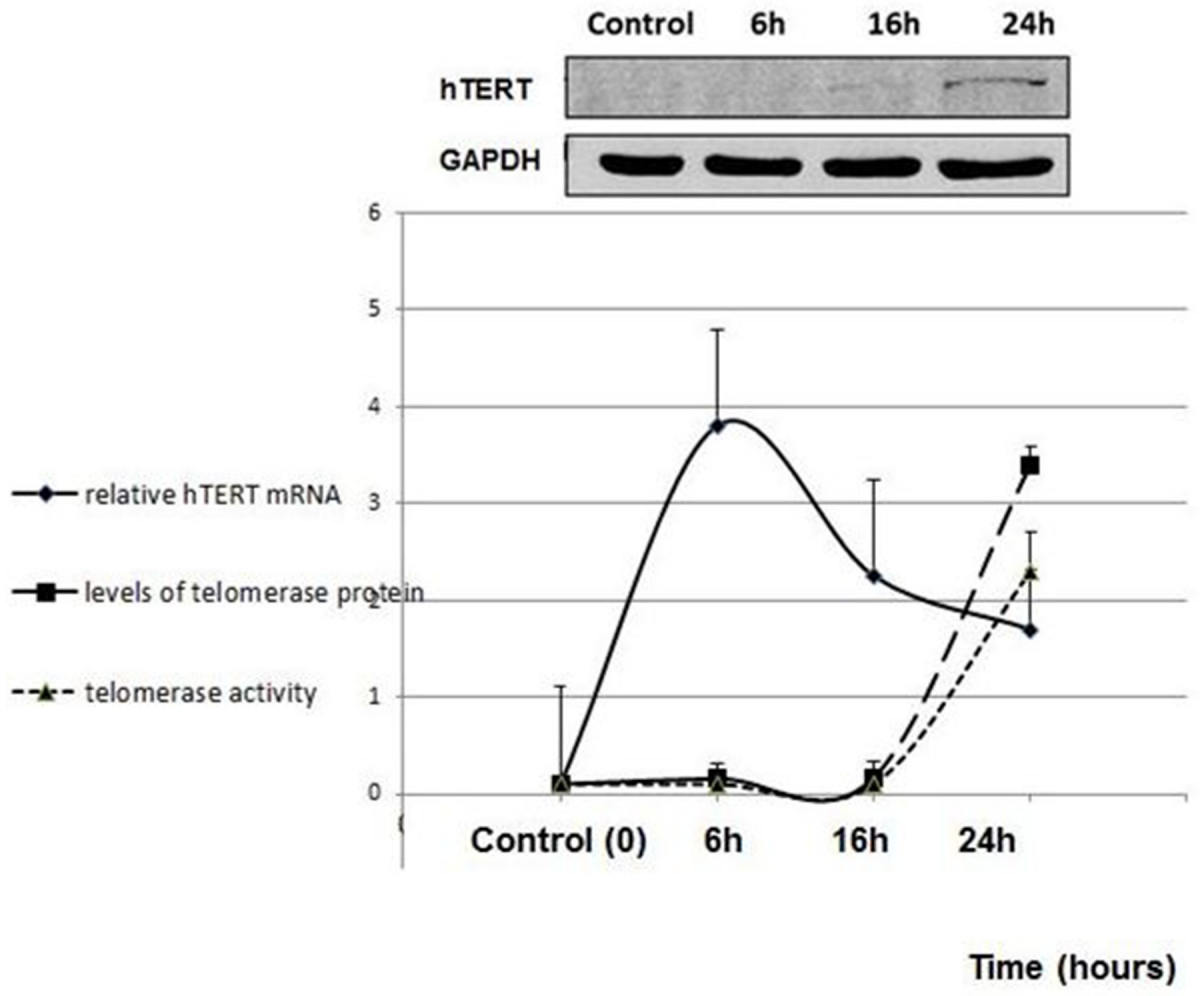
*De novo* telomerase activity in pHFF cells following Jurkat cells derived exosomal exposure pHFF cells were subjected to Jurkat-derived exosomes for 6, 16 and 24 hrs. Cells were harvested, lysed and analyzed for the expression of the hTERT mRNA by quantitative real time PCR; telomerase protein levels by Western blotting and telomerase activity by Q-TRAP assay. At the upper panel of the figure a representative image of the Western blot analysis is shown. The graphs represent average values of each sample ± S.E of three or more independent experiments conducted in triplicates.

Like the majority of somatic cells fibroblasts do not exhibit telomerase activity due to transcriptional repression of hTERT. pHFF cells (primary non-cancer human fibroblasts) were exposed to Jurkat cells derived exosomes. Isolated exosomes were labeled by an acridine orange based die that binds the RNA content of exosomes. Six hours post exposure to exosomes, a remarkable uptake of the secreted exosomes by the fibroblasts cells was observed (Figure 5). Concomitant with exosomal uptake the levels of recipient intracellular hTERT mRNA substantially increased (Figure 6) reaching a maximum 6 hr post exposure. As shown in Figure 6 the level of telomerase protein and its activity increased more than 3 fold 24 hours post incubation with exosomes. Notably, these fibroblasts did not further secrete hTERT mRNA in their exosomes (not shown).

The results show that the hTERT mRNA delivered by cancer-cells derived exosomes into telomerase-null recipient cells is translated to a fully active enzyme; telomerase activity was detected together with an increase in the level of protein.

### Telomerase shuttle via Jurkat cells derived exosomes markedly increases proliferation and life span of the recipient fibroblast cells

**Figure 7:**
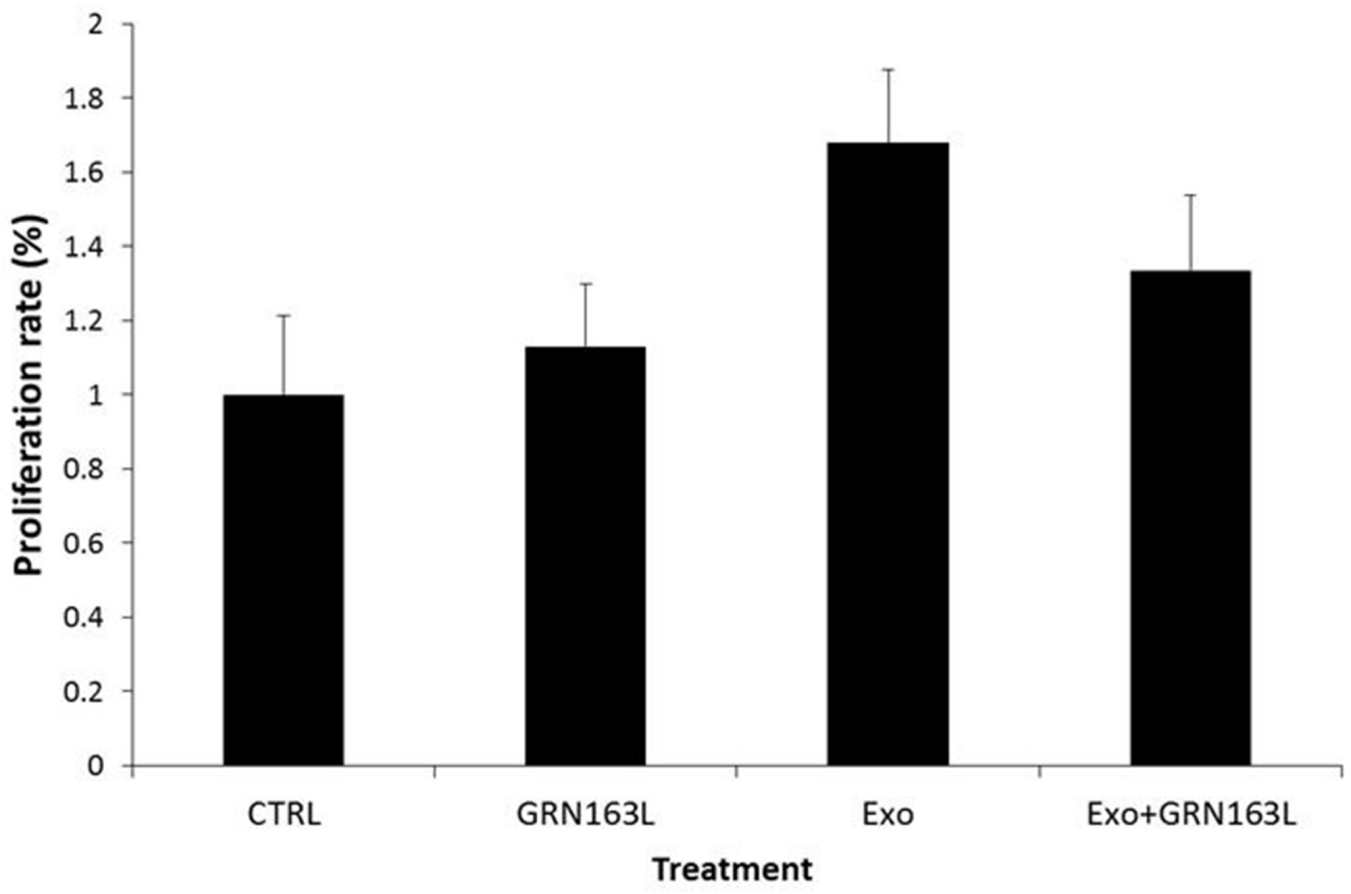
Proliferation rates in response to *de novo* telomerase activity in pHFF cells pHFF cells were subjected to Jurkat-derived exosomes (Exo), to telomerase inhibitor (GRN) or to Jurkat-derived exosomes and telomerase inhibitor (Exo+ GRN) twice a week for five weeks. The proliferation rate of the cells was measured by the Trypan blue exclusion assay.

After demonstrating *de novo* telomerase activation in the recipient cells we assessed the effects of this phenomenon on the proliferation of the recipient cells. For that purpose pHFF cells were exposed to Jurkat cells derived exosomes with or without telomerase inhibitor, GRN 163 for several weeks. Remarkably, cells exposed to exosomes harboring the hTERT mRNA exhibited higher proliferation rate already one week post exosomal exposure (Figure 7). Additional exposure of these cells resulted in elongation of their life span. Whereas the control untreated cells died after 36 population doublings (PD), the cells exposed to exosomes continued to proliferate beyond PD 42. These effects were telomerase dependent to some extent, as cells in which telomerase was inhibited showed a much milder effect (Figure 7). Together, these results suggest that hTERT mRNA shuttling to primary fibroblasts affect the biology of the recipient cells, e.g. proliferation and life span.

### Telomerase shuttle via Jurkat cells derived exosomes protects from late senescence

**Figure 8:**
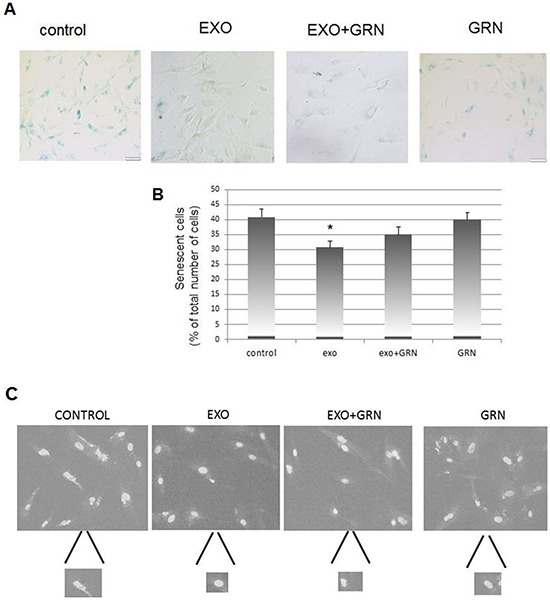

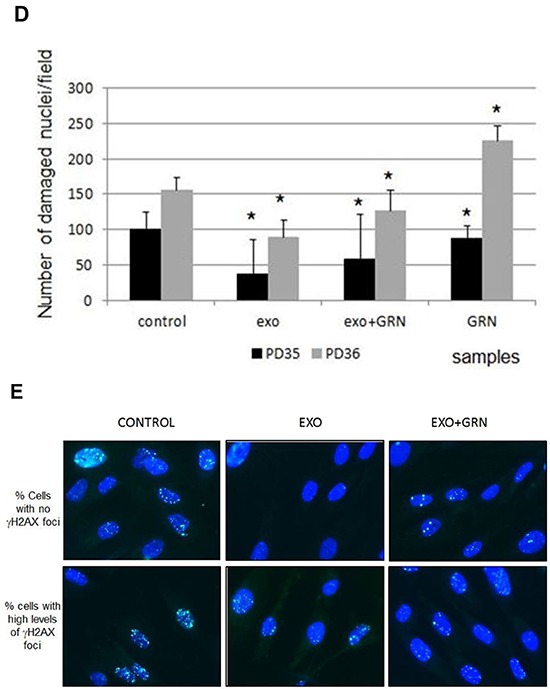

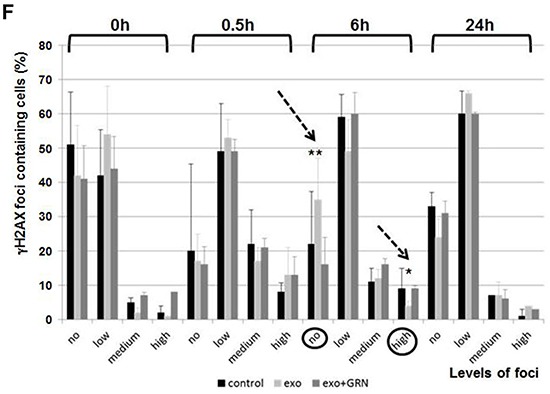
Phenotypic changes in response to *de novo* telomerase activity in pHFF cells pHFF cells were subjected to Jurkat-derived exosomes (Exo), to telomerase inhibitor (GRN) or to Jurkat-derived exosomes and telomerase inhibitor (Exo+ GRN) twice a week for five weeks. The following parameters were analyzed: cellular senescence, appearance of nuclei morphology and DNA damage response. **A.** Effects of *de novo* telomerase activity on senescence of pHFF cells. pHFF cells were grown in the presence of exosomes and senescence was assayed by the β-Galactosidase activity assay. Blue staining demonstrates β-Galactosidase activity. For each treatment 10 fields were captured under the microscope and counted. Representative pictures are shown. **B.** Quantification of β-Galactosidase activity assay. * Indicates P<0.05. **C.** Effects of *de novo* telomerase activity on the morphology of pHFF nuclei. pHFF cells were grown as above and stained with DAPI. Damaged nuclei were inspected. For each treatment 10 fields were captured under the fluorescent microscope, damaged and total number of nuclei was determined. Represented images are shown. **D.** Quantification of irregularity of nuclei. * Indicates P<0.05. **E.** Effects of de novo telomerase activity on the repair of DNA damage imposed by phleomycin on pHFF cells. pHFF cells were exposed once to Jurkat derived exosomes for three days. Cells were than exposed to 20 ug/ml of phleomycin for 0.5, 6 and 24 hours, stained for γH2AX foci by immunofluorescent antibody and for DNA by DAPI. Treatments were conducted in triplicates and for each treatment six fields were captured under a fluorescence microscopy. γH2AX foci were counted and the numbers were divided to four levels: no foci (o), low (1-10), medium (11-20) and high (20- countless) number of foci. Representative images of cells six hours post phleomycin treatment are shown. **F.** Quantification of E. Arrows point to two samples in which significant differences were calculated with regards to cells exposed to exosomal hTERT versus control cells or cells in which telomerase was inhibited. Each bar represents the average and standard error results of three independent experiments. * Indicates P<0.05, ** Indicates P<0.001.

Same pHFF cells were exposed to Jurkat cells derived exosomes with or without telomerase inhibitor, GRN 163 for several weeks as above. β-galactosidase staining reviled that control untreated cells entered senescence state earlier than those exposed to Jurkat cells derived exosomes (Figure 8A, 8B). This protection from senescence is probably telomerase dependent to some degree, as cells in which telomerase was inhibited showed a much milder effect (Figure 8).

Inspection of the nuclei of these cells revealed a change in the morphology manifesting in nuclear irregularity of control cells that reached late senescence. This change was not detected in cells exposed to exosomes. Some of the nuclei exposed to exosomes in which telomerase was inhibited exhibited changes in their morphology, indicating an intermediate situation (Figure 8c, 8d). The fact that telomerase inhibition in cells exposed to cancer derived exosomes fails to abrogate completely the anti- senescence effect suggests that this effect may be mediated also by additional factors.

### Telomerase shuttle via Jurkat cells derived exosomes protects from DNA damage

Next we were interested to evaluate whether exosomal hTERT is capable of executing one of non-telomere related activities attributed to telomerase: protection from DNA damage. pHFF cells were exposed to Jurkat derived exosomes with or without telomerase inhibitor for three days. Cells were then exposed to 20 ug/ml of phleomycin for 0.5, 6 and 24 hours and stained for γH2AX foci and DNA analysis (by immunofluorescence). Similar amounts of γH2AX foci were obtained after exposure to exosomes, before, 0.5h and 24 hours post phleomycin addition. However, six hours after DNA damage insult there was a difference between cells exposed to exosomes and control cells or those in which telomerase activity was inhibited: there were many more cells which contained no γH2AX foci and similarly there where less cells with high levels of these γH2AXfoci in cells exposed to exosomes (Figure 8E, 8F). These results indicate that exosomal hTERT uptake in fibroblasts protects from DNA damage induced in the cells.

## DISCUSSION

Recently, the involvement of exosomes in the crosstalk between malignant cells and the tumor microenvironment has been documented [28]. The current study demonstrates for the first time that hTERT mRNA, the transcript of the enzyme telomerase, is shuttled from “donor” cancer cells via exosomes into “recipient” telomerase negative fibroblasts, where it is translated into a fully active enzyme. All tested cancer cells secreted exosomal hTERT mRNA in accordance with the endogenous levels of their hTERT mRNA and telomerase activity. It is well known that various cancer types differ in the level of their telomerase activity [29]. The correlation between telomerase activity in cancer cells and their exosomes may be of clinical importance also in another aspect as a biomarker in cancers which prognosis is correlated with the level of telomerase activity [30].

Although hTERT mRNA was previously mentioned in a list a various mRNAs in exosomes secreted from glioblastoma cells [31], our findings are novel since it is the first study describing exosomal hTERT mRNA secretion and its fate.

The dynamics of exosomal hTERT mRNA uptake and translation by fibroblasts show six hours for uptake and 24 hours to translation to active telomerase. The downregulation of the transcript after six hours imply that the source of hTERT mRNA is indeed exogenous to the fibroblasts. In the human body the secretion and uptake of exosomes is probably a continuous process, therefore we would expect a stable telomerase activity and its subsequent effects in the recipient cells will be more pronounced. The demonstration of exosomal hTERT mRNA in serum from patients with cancer suggest that our *in vitro* model is relevant to clinical setting.

The phenomenon of exosomal nucleic acids taken up by cells and translated in the recipient cells has been described previously in a mouse model [31]. However transformation of telomerase negative somatic cells into telomerase positive has never been reported. As known, somatic human cells are inherently telomerase negative and this negativity determines many aspects of cellular biology (such as senescence, limited life span and other properties). Therefore, their transformation may have profound implications on various aspects of their biology and some of these changes are shown in this study.

Overexpression of telomerase in initially telomerase negative cells was shown to affect their biological properties. These cells exhibited markedly extended or unlimited life span, prevention and sometimes reversal of senescence– both resulting from telomere length maintenance [32]. Telomerase overexpression results also in protection from DNA damage, prevention of apoptosis in certain conditions, activities unrelated to telomere length and termed “extracurricular” activities of telomerase [33]. Several of these aspects were examined in this study.

In order to be active, telomerase enzyme undergoes several post-translational regulations including assembly to the RNA unit, hTR, and to other related proteins and to be phosphorylated [34]. Although we did not directly assessed whether this assembly indeed took place in the fibroblasts, the fact that phenotypic changes known to be causes by telomerase activity after exosomal uptake were observed suggests that the enzyme was correctly assembled.

Primary fibroblast cells exposed to exosomes for five weeks exhibited increase in their proliferation rate. Other studies have also shown that fibroblast or endothelial cells exposed to cancer derived exosomes increase their rate of proliferation [35]. We show that this effect was at least partially caused by induction of telomerase activity, since it was attenuated by the addition of telomerase inhibitor. The effect of telomerase on cellular proliferation is controversial. Some studies suggest that telomerase promotes the proliferation rate (e.g. 36, 37) while other studies claim that proliferation is not dependent or influenced by telomerase activity [38]. The results of this study may lend support to the first scenario [39–41].

Another effect noted in this study is the extension of cellular life span. This effect also has been reported upon transfection of telomerase gene [42]. The exosomal hTERT did not confer immortality on the fibroblasts, probably due to the level of telomerase activity which was lower compared to that of gene overexpression. In addition, exosomes were applied every 3-4 days and not continuously. It is plausible that in *in vivo* conditions the cancer surrounding fibroblasts may acquire significant increase of life span bordering on immortality.

Exosomal telomerase transfer also postponed the cellular senescence as expected in cells with telomerase activity [43]. We used two methods for characterization of senescence: the widely accepted measurement of β-galactosidase activity and the differences in the appearance of nuclear architecture [44–46].

Protection from DNA damage has been reported as an “extracurricular” activity of telomerase. Indeed, the “telomerase activated” fibroblasts were protected from phleomycin induced DNA damage, protection that was abrogated by telomerase inhibition. This was demonstrated six hours after phleomycin exposure, where a statistically significant change between the percentages of cells exposed to exosomal hTERT versus same cells treated also with telomerase inhibitor with no detected DNA damage (represented by the number of γH2AX foci) was observed. The results regarding percentage of cells with high numbers of γH2AX foci were not necessarily significant biologically, although reached a statistical significance.

The cellular effects described in our paper are similar to the changes in telomerase negative cells after transfection with telomerase open reading frame [46]. We surmise that *in vivo*, in tumor microenvironment the effects of telomerase activation will be more pronounced due to the continuous manner of exposure, while in our study the exosomes were added only every three days.

In summary, we demonstrate here another aspect of exosomal mediated cancer to non-cancer cell crosstalk. Transformation of telomerase negative to telomerase active is a fundamental change in cell's biology. In fact it leads to formation of a novel type of cells; non malignant cells with active telomerase. The implications of this process may be wide ranging and deserves further studies. Specifically, this process may contribute to the unique aspects of tumor microenvironment including formation of cancer associated fibroblasts (CAF).

Limitations of this study derive from the experimental setting being mostly *in vitro*. The exosomes were applied non continuously and therefore the results might be much more pronounced in a situation in which there is a continuous exosomal transfer such as co-culture model (currently set up in our laboratory).

The mechanism of packaging of hTERT in exosomes is not clear, except a general description of the involvement of the ESCRT complex in packaging nucleic acids in exosomes [47]. Clearly the content of exosomes is highly regulated and future studies should be directed at deciphering the mechanisms underlying the packaging process and its importance.

Further studies will explore the relevance of the cellular changes described in this paper and their effects on tumor microenvironment. Understanding of the biological process and the therapeutic potential for their inhibition is a promising research avenue.

## MATERIALS AND METHODS

### Experimental system

Jurkat and K562 cell lines were cultured in RPMI-1640 supplemented with 20% and 10% fetal bovine serum (FBS), respectively, containing 100 units/ml L-glutamine and 1% penicillin/streptomycin (Biological Industries Beit Haemek, Israel). MCF-7, HCT-116 and pHFF (primary human foreskin fibroblasts, kindly provided by Sara Selig, the Technion - Israel Institute of Technology, Israel) cell lines were cultured in DMEM with 10% FBS supplemented with the same supplements as mentioned for Jurkat and K562 cells under standard incubation conditions. Prior to isolation of exosomes from the cells' growth media, the cells were transferred to exosome depleted media to prevent contamination with FBS exosomes. 10 ml serum was obtained from 5 patients with pancreatic and lung cancer. The patients signed an informed consent for the study that was approved by IRB.

### Ultracentrifugation

Depletion of exosomes from FBS was executed via ultracentrifugation of the FBS at 100,000g for 16 hours at 4°C (Optima™ XPN, Beckman coulter).

### Isolation of exosomes

Exosomes were extracted from either cell culture media or patients' serum by using the miRCURY™ Exosome Isolation kit (Exiqon, MA, USA) according to the manufacturer's instructions. Briefly, isolation reagent was added to cell culture media and incubated at 4°C for 16hr. The precipitated exosomes were recovered by centrifugation at 10,000 x g for 60 min. The pellet was then resuspended in PBS and the isolated exosomes were analyzed immediately or after storage at −80°C. Exosomes were inspected by using the NanoSight device (NanoSight, Amesbury, UK).

### Exposure of fibroblasts to exosomes

Cancer cells were incubated for 72hr in exosome depleted media in order to collect Jurkat derived exosomes. The media was centrifuged twice at 2,000g for 30 min to remove cells and debris and then incubated for 16hr with the exosome isolation reagent. In the following day, the media was centrifuged at 10,000g for one hour and the isolated exosomes were resuspended in pHFFs culture. 200μl of the resuspended exosomes were added to 3*10^5^ pHFF cells per well in a 6-well culture plate and the cells were harvested after 6, 16 and 24 hours. The cells were divided into two aliquots: one was used to isolate RNA for the hTERT expression assay by q-PCR and the other was used to isolate proteins for estimation of telomerase activity by the q-PCR based TRAP assay and the level of telomerase by Western blot. The concentration of exosomes was about 150 microgram per ml culture. For longer exposure (several weeks) of fibroblasts to exosomes performed for other analyses such as population doubling, senescence and apoptosis, pHFF cells were exposed to Jurkat derived exosomes twice a week at the above conditions.

### Exosome uptake validation assay

Isolated exosomes were labeled with Exo-Glow exosome labeling kit (System Bioscience, CA, USA) according to the manufacturer's instructions. Briefly, 50 μl of 10x Exo-Red were added to 500 μL of resuspended exosome in 1x PBS and incubated at 37°C for 10 minutes. The labeling reaction was stopped by adding 100 μl of the ExoQuick-TC reagent and incubated on ice for 30 min. The labeled exosomes were centrifuged, resuspended in 1xPBS and added to 1*10^5^ pHFF cells per well in a 6-well culture plate. The cells with the labeled exosomes were incubated for 2 to 24 hours and visualize by fluorescent microscopy after DAPI staining (GenASIs Scan, Applied Spectral Imaging, Israel).

### RNA isolation

RNA extraction from cells was performed using the EZ-RNA II Isolation Kit (Biological Industries Beit Haemek, Israel) according to the manufacturer's instructions. Extracted RNA was quantified by the NanoDrop spectrophotometer with ND-1000 software (Thermo scientific, MA, USA). The RNA extraction from exosomes was executed using the Total Exosome RNA and Protein Isolation Kit (Invitrogen, CA, USA) according to manufacturer's instructions.

### cDNA synthesis

Total RNA extracts from cells and exosomes were reverse transcribed using the High-Capacity cDNA Reverse Transcription Kit (Applied Biosystem, CA, USA) according to the manufacturer's instructions.

### hTERT expression by Real Time PCR (q-PCR)

The expression of the *hTERT* gene was measured by q-PCR. PCR reactions were prepared with custom made TaqMan fluorochrome labelled primers (Applied Biosystems, CA, USA).

hTERT primers: Forward: 5′-CGTCCAGACTCCGC TTCATC-3′

Reverse: 5′-GAGACGCTCGGCCCTCTT-3′;

HPRT-1 primers: Forward: 5-′TTATGGACA GGACTGAACGTCTTG-3′

Reverse: 5′-TGTAATCCAGCAGGTCAGCAAA-3′.

### Sequencing of hTERT PCR product

Sequencing was performed on the PCR product of hTERT using the same primers. 3*10^5^ cells/ml of Jurkat cells were incubated for 72hr in exosome depleted media in order to isolate Jurkat derived exosomes. RNA was extracted from the isolated exosome, reverse transcribed to cDNA and amplified using the 2XReddyMix PCR Master Mix (Thermo Scientific, MA, USA) according to the manufacturer's instructions. Briefly, 10μM of forward and reverse primers were added to a mix reaction including 2XReddyMix PCR Master Mix, 125 ng of template DNA and PCR grade water. Incubation steps included initial denaturation at 95°C for 2 min followed by 40 cycles of 95°C for 25 sec, 60°C for 35 sec and 72°C for 65 sec. 2μl of the PCR product were separate on a 2% agarose gel in order to purify the PCR product and to verify its size and specificity. 20ng/μl of the DNA template was added to four different reactions containing 5 pmol/μl of each of the above primers. Sequencing reactions were performed by HyLabs (HyLabs, Rehovot, Israel). Basic Local Alignment Search Tool (BLAST) software was employed in order to verify the annotation of the PCR products (BLAST software, NCBI).

### q-TRAP assay

Telomerase activity was determined by the TRAP assay using the q-PCR based telomerase detection kit (Allied Biotech Inc., CA, USA), according to the manufacturer's instructions. Briefly, cells were lysed and protein concentration was determined by the Bradford assay (Bio-Rad Laboratories, CA, USA). 500ng protein extract was taken for each 25μl reaction containing telomeric oligonucleotides, dNTPs, reaction buffer and SYBR green. The extended products were subsequently amplified by q-PCR. Generated PCR products were then visualized using SYBR Green, measuring the increase in fluorescence resulted from the intercalation of the SYBR Green dye into the double-stranded DNA. Telomerase activity was then quantified by a calibration curve of known quantities of TSR segments.

### Western blotting

Verification of the extracted exosomes was determined by Western blotting using antibodies against well-characterized exosomal protein markers: CD63, CD9 and TSG101. Extracted exosomes were resuspended in RIPA buffer, sonicated and quantified using Pierce BCA Protein Assay Kit (Thermo Scientific, MA, USA). 50μg of protein was subjected to 10% Sodium Dodecyl Sulfate Poly Acrylamide gel electrophoresis (SDS-PAGE) and transferred to a nitrocellulose membrane. The membrane was then hybridized for 16h at 4°C with antibodies against exosomal markers: anti-CD63, anti-CD9 (1:1000, Santa Cruz Biotech, TX, USA) and anti-TSG101 (1:500, Abcam MA, USA). In the following day the membrane was subjected to fluorescent labeled secondary antibodies. Visualization was done by the Odyssey analysis software (Odyssey IR imaging system; LI-COR).

The levels of telomerase in pHFF cell line were evaluated by Western blotting as well. The cells were grown for 72hr in the presence of Jurkat derived exosomes. 50 μg of protein were separated by 10% SDS-PAGE, transferred to nitrocellulose membrane and hybridized for 16h at 4°C with specific antibodies: anti-telomerase (1:500, Abcam, MA, USA) Signals were visualized after exposing the membranes to 2^nd^ fluorescent antibodies and quantified by the Odyssey analysis software (Odyssey IR imaging system; LI-COR).

### Proliferation assay

Proliferation of fibroblasts was measured by the Trypan blue exclusion method which distinguishes between apoptotic or necrotic cells and live cells. 5μM GRN163, a potent telomerase inhibitor (48), generously provided by Geron Corporation (Menlo Park, CA) was used in order to prove the involvement of telomerase *per-se* in the proliferation effects.

### Population doubling (PD)

PD was calculated according to the following formula: #PD= log of number of harvest cellsX3.3218-log of number of plated cellsX3.3218.

### DNA damage

pHFF cells were exposed to Jurkat derived exosomes with or without telomerase inhibitor for three days. Cells were than exposed to 20 ug/ml of phleomycin for 0.5, 6 and 24 hours and stained for γH2AX foci and DNA analysis by immunofluorescence as previously described [49]. Each treatment was performed in triplicates. The foci were counted in each slide (6 fields per slide) and were divided to four levels: no foci (o), low (1-10), medium (11-20) and high (countless) number of foci.

### Senescence- associated β-galactosidase

pHFF cells were exposed to Jurkat derived exosomes with or without telomerase inhibitor twice a week for five weeks. Cells were analyzed every week for -galactosidase activity by using the β-Galactosidase Detection kit (abcam, Cambridge, UK) according to the manufacturer's instructions. Briefly, cells were washed, fixed and stained with the X-Gal reagent followed by DAPI staining for the detection of cell nuclei as described previously [50]. For each treatment blue- stained cells were visualized under the fluorescent microscope (GenASIs Scan, Applied Spectral Imaging, Israel), counted and divided to the total cell number in each field. Ten fields were analyzed for each treatment.

### Nuclear morphology

Cells were treated as described above for senescence assay. Nuclei with different morphology were visualized under the fluorescent microscope (GenASIs Scan, Applied Spectral Imaging, Israel), counted and divided to the total number of nuclei in each field (10 fields per treatment).

### Statistical analysis

A two-tailed Student T Test with unequal variance and one way ANOVA were used to calculate the *P* values in SPSS for Windows version 11.5 software (SPSS, Inc., Chicago, IL). All results are shown as average ± standard error (SE). In all assays, *P* values of less than 0.05 were considered statistically significant.
